# iAPSL-IF: Identification of Apoptosis Protein Subcellular Location Using Integrative Features Captured from Amino Acid Sequences

**DOI:** 10.3390/ijms19041190

**Published:** 2018-04-13

**Authors:** Yadong Tang, Lu Xie, Lanming Chen

**Affiliations:** 1 Key Laboratory of Quality and Safety Risk Assessment for Aquatic Products on Storage and Preservation (Shanghai), China Ministry of Agriculture, College of Food Science and Technology, Shanghai Ocean University, Shanghai 201306, China; m150208365@st.shou.edu.cn; 2 Shanghai Center for Bioinformation Technology, Shanghai Academy of Science and Technology, Shanghai 201203, China

**Keywords:** apoptosis proteins, Markov chains, physicochemical properties, position specific scoring matrix, support vector machine, recursive feature elimination

## Abstract

Apoptosis proteins (APs) control normal tissue homeostasis by regulating the balance between cell proliferation and death. The function of APs is strongly related to their subcellular location. To date, computational methods have been reported that reliably identify the subcellular location of APs, however, there is still room for improvement of the prediction accuracy. In this study, we developed a novel method named iAPSL-IF (identification of apoptosis protein subcellular location—integrative features), which is based on integrative features captured from Markov chains, physicochemical property matrices, and position-specific score matrices (PSSMs) of amino acid sequences. The matrices with different lengths were transformed into fixed-length feature vectors using an auto cross-covariance (ACC) method. An optimal subset of the features was chosen using a recursive feature elimination (RFE) algorithm method, and the sequences with these features were trained by a support vector machine (SVM) classifier. Based on three datasets ZD98, CL317, and ZW225, the iAPSL-IF was examined using a jackknife cross-validation test. The resulting data showed that the iAPSL-IF outperformed the known predictors reported in the literature: its overall accuracy on the three datasets was 98.98% (ZD98), 94.95% (CL317), and 97.33% (ZW225), respectively; the Matthews correlation coefficient, sensitivity, and specificity for several classes of subcellular location proteins (e.g., membrane proteins, cytoplasmic proteins, endoplasmic reticulum proteins, nuclear proteins, and secreted proteins) in the datasets were 0.92–1.0, 94.23–100%, and 97.07–100%, respectively. Overall, the results of this study provide a high throughput and sequence-based method for better identification of the subcellular location of APs, and facilitates further understanding of programmed cell death in organisms.

## 1. Introduction

Apoptosis, or programmed cell death, is a fundamental process controlling normal tissue homeostasis by regulating the balance between cell proliferation and death [1]. Blocking apoptosis is associated with cancer and autoimmune diseases (e.g., autoimmune lymphoproliferative syndrome (types I and II) and systemic lupus erythematosus), whereas unwanted and increased apoptosis can lead to ischemic damage or neurodegenerative diseases (e.g., Alzheimer’s disease, Parkinson’s disease, amyotrophic lateral sclerosis, and Creutzfeldt-Jakob disease) [2,3,4]. The subcellular location (e.g., membrane, cytoplasm, nuclear, endoplasmic reticulum, and mitochondria) of APs is strongly related to their function [2]. Subcellular location can be identified using conventional experimental methods, such as electronic microscopy, cell separation, and fluorescence microscopy [5]. Nevertheless, these experimental methods are time-consuming and expensive [5]. Facing the explosion of new protein sequences generated in the post-genomic and big data age [6], there exists a clear need for developing high-throughput, and sequence-based methods to identify the subcellular location of APs.

**Figure 1:**
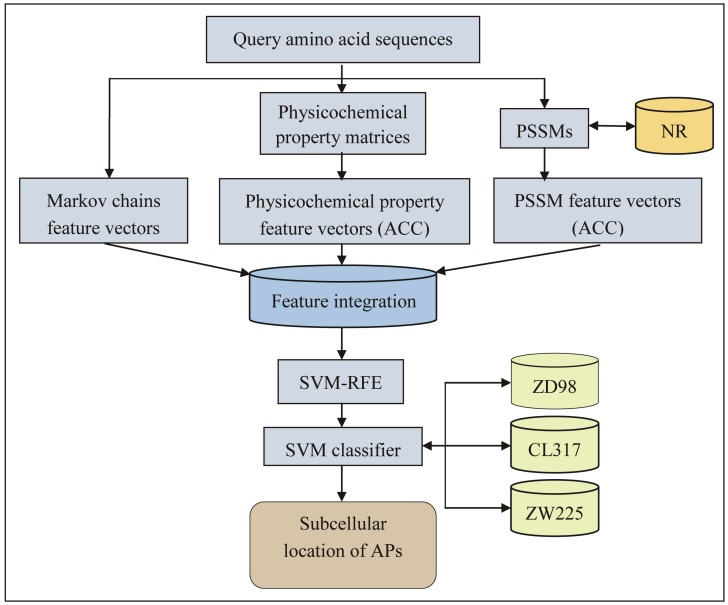
The flowchart of the iAPSL-IF method. NR: non-redundant (NR) database of the National Center for Biotechnology Information (NCBI) (available online: https://www.ncbi.nlm.nih.gov/).

To date, computational methods have been reported to efficiently identify the subcellular location of APs [7]. These methods were developed based on; (1) the design of the protein encoding scheme of the feature extraction; (2) the selection of the classifier [7]. Some sequence features are used for the first task, e.g., amino acid composition [8], dipeptide composition, which represents the composition of amino acid pairs and gapped amino acid pairs [9], pseudo amino acid composition [10,11,12,13,14], Markov chains [15], wavelet coefficients [3], distance frequency [16], grouped weight encoding [2], PSSMs [7,17,18], and gene ontology [19,20]. For example, the Markov chains, being a discrete stochastic model [21], contain the frequencies of 20 native amino acids and the information of amino acid pairs in protein sequences, which reflect the composition and local amino acid order of the protein sequences. They have been used for the identification of interaction sites between proteins and nucleic acids [21,22]. The PSSM reflects the evolutionary information of a protein sequence, and has been used for the prediction of protein function [23], subcellular location [5], and structural class [24,25]. In addition, a few machine learning algorithms have been developed for the second task, including the fuzzy *k*-nearest neighbor algorithm [12], SVM [3,7,16,17,18], covariant discrimination algorithm [9], and ensemble classifier [26,27]. Among these, the SVM proposed by Vapnik [28] exhibited the most promising results [7]. It is a supervised machine learning algorithm based on the structural risk minimization principle of statistical learning theory [26]. Samples labeled positive or negative are projected into a high dimensional feature space using a kernel, in which the hyper plane is optimized to maximize the margin of positive and negative samples [29]. For the SVM-based methods, it is crucial to convert the protein sequences with different lengths into fixed-length vectors [18]. The ACC transformation method was developed by Wold et al. [30], and has been widely used in protein family classification and protein interaction prediction [31,32]. Although computational methods, such as PSSM-trigram [7] and FKNN (fast k-nearest neighbor algorithm) [12], have been reported to reliably identify the subcellular location of APs, there is still room for improvement of the prediction accuracy. In our previous research, we established highly accurate protein structural class prediction methods based on the PSSMs using the SVM classifier [25,32]. In this study, we developed a novel method named iAPSL-IF using integrative features captured from amino acid sequences (Figure 1), and examined it based on three datasets ZD98, CL317, and ZW225 using the jackknife cross-validation test, as it is an objective and rigorous statistical test [22]. In jackknifing, a part of the sample is systematically omitted, for example, by removing one data point at a time, and the analysis is then carried out for each newly constructed subset [33]. Our data indicated that the iAPSL-IF achieved better results than the known predictors reported in the literature.

## 2. Results and Discussion

### 2.1. Feature Extraction

In order to capture the feature information embedded in amino acid sequences, we analyzed amino acid compositions and Markov chains of each protein sequence in the three datasets ZD98, CL317, and ZW225, and encoded each sequence by a 420 (20 × 20 + 20) dimensional feature vector (see the Materials and Methods section). Meanwhile, the 10 physicochemical properties of amino acids were also individually numbered based on their corresponding values [34] for these protein sequences, and then each sequence was replaced by a numerical physicochemical property matrix. Furthermore, the evolutionary information of these protein sequences was each extracted by BLAST analysis, and then each sequence was represented by a PSSM. The resulting physicochemical property matrices and PSSM displayed different lengths, based on the different protein sequences.

### 2.2. Parameter Selection

**Figure 2:**
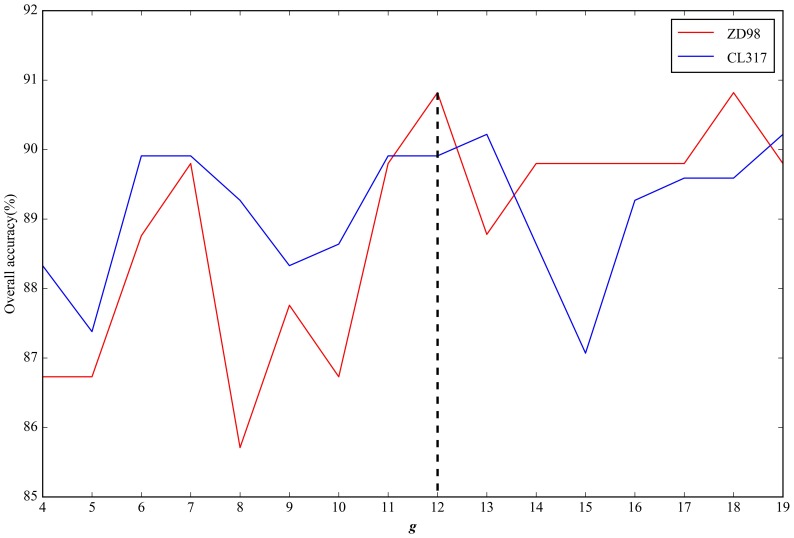
The effects of *g* on overall accuracy based on the datasets ZD98 and CL317 after ACC transformation of the physicochemical property matrices.

**Figure 3:**
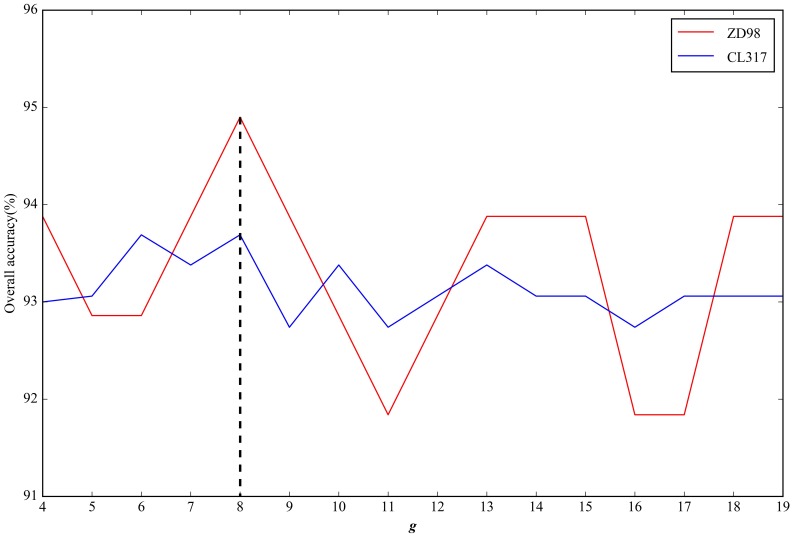
The effect of *g* on overall accuracy based on the datasets ZD98 and CL317 after ACC transformation of the PSSMs.

To transform the physicochemical property matrices and PSSMs with different lengths into fixed-length feature vectors using the ACC method, we analyzed the key parameter length (*g*). The *g* values were set in the range of 4 ≤ *g* ≤ *L*/4 [17], where *L* is the length of the shortest protein sequence in a dataset. For the three datasets ZD98, CL371, and ZW225, *L* was 130, 87, and 76, respectively. We used the jackknife cross-validation test to measure the overall accuracy of the datasets corresponding to different *g* values. The resulting data were illustrated in Figure 2 and Figure 3. For the physicochemical property matrices, the highest overall accuracy of the datasets ZD98 and CL317 were 90.82% and 90.22%, when *g* = 12 and 13, respectively (Figure 2). In order to guarantee that the dimensions of the vectors were consistent, we set *g* = 12 for the ACC transformation of the physicochemical property matrices. Hence, each protein sequence was encoded by a 1100 (10 × 10 × (12 − 1)) dimensional vector. Likewise, for the PSSMs, when *g* = 8, the highest overall accuracy of the datasets ZD98 and CL317 were observed (94.90% and 93.69%, respectively). Therefore, each protein sequence was also replaced by a 2800 (20 × 20 × (8 − 1)) dimensional vector through the ACC transformation (Figure 3). Given the same dimension (420) for the Markov chains vectors, we obtained a 4320 (1100 + 2800 + 420) dimensional vector for each protein sequence by integrating the three different types of sequence features. Similarly, the parameter based on the dataset ZW225, with a similar size as CL371, was also determined.

### 2.3. Optimal Feature Selection

**Figure 4:**
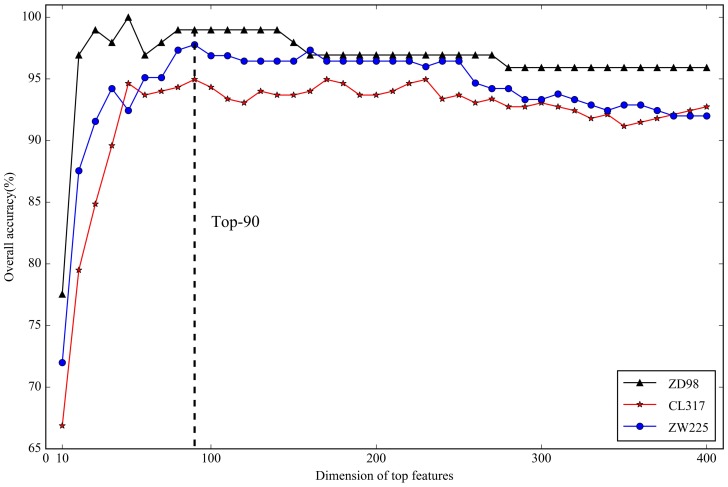
The effect of the Top-*K* features on overall accuracy based on datasets ZD98, CL317, and ZW225.

Although the integrated features captured sequence information from multiple aspects, the number of candidate features was large and the original feature space may have contained noisy and redundant features. Therefore, we reduced the dimensions using the SVM-RFE method [35], and improved the performance: (1) less prone to overfitting; (2) able to make full use of the training data; (3) much faster [29]. The feature vectors of a dataset were ranked according to their importance, and their top-*K* (*K* = 10, 20, 30, …, 380, 390, 400) [32] features were examined by the jackknife cross-validation test. The resulting data were illustrated in Figure 4. Based on the dataset ZD98, when *K* = 50, the highest overall accuracy (*OA*) was observed (100%), whereas when *K* = 90, the highest *OA* was 94.95% and 97.78% on the datasets CL317 and ZW225, respectively. In order to avoid losing important information if the dimension was low, we choose the top-90 ranked features for further analyses.

### 2.4. Performance of the iAPSL-IF

In this study, each protein sequence was encoded by a 90-dimensional vector after feature integration and optimal feature selection. We trained these features using the SVM and developed the iAPSL-IF. The performance of the iAPSL-IF was examined by the jackknife cross-validation test based on the three datasets, and the results were presented in Table 1. Based on the datasets ZD98, CL317, and ZW225, the *OA* was 98.98%, 94.95%, and 97.33%, respectively; the sensitivity (*S_ens_*) for different classes of subcellular location proteins was 97.67–100%, 88.24–100%, 88.00–100%, respectively; the specificity (*S_pec_*) was 98.53–100%, 97.07–100%, 98.71–100%, respectively; and the Matthew’s correlation coefficient (*MCC*) was 0.98–1.00, 0.88–0.99, 0.90–0.98, respectively. Notably, among the seven classes of subcellular location proteins tested in this study, only the *S_ens_* of the mitochondrial proteins (Mito) in the datasets CL317 and ZW225 was slightly lower (88.24% and 88.0%, respectively) than the other subcellular location proteins. Moreover, their corresponding *MCC* values were also lower (0.88 and 0.90, respectively). Similar results yielded by some previous predictors were also reported [1,10,23]. This may result from the discrepancies in dataset traits, such as the size, sequence homology, and unbalance of the subsets [16].

### 2.5. Performance Comparison with Other Known Methods

To evaluate how reliable the performance of the iAPSL-IF was, we compared it with all the known methods based on the same datasets available in the literature. The *OA* and *S_ens_* of different subcellular location proteins were chosen as the evaluation indexes for the jackknife cross-validation test. Based on dataset ZD98, the *OA* was 76.5–96.9%, as identified by twelve previous predictors, among which, the PSSM-trigram had the best performance (96.9%). However, the iAPSL-IF developed in this study further increased the *OA* by 2.1% when compared with the PSSM-trigram (Table 2). Moreover, the highest *S_ens_* value was also achieved by the iAPSL-IF for the cytoplasm proteins (Cyto) (97.7%), membrane proteins (Memb) (100%), Mito (100%), and other proteins (100%) (Table 2).

Based on dataset CL317, the performance of the iAPSL-IF was compared with ten known predictors by the jackknife cross-validation test. The *OA* of the predictors ranged between 82.7% and 95.0%, among which the iAPSL-IF achieved the best performance (95.0%) with an increase of 2.6%. Although the *S_ens_* values for the Cyto, Memb, and Mito proteins identified by the iAPSL-IF were slightly lower (95.5%, 94.5%, 88.2%) than the previous better predictors (81.3–99.1%, 81.8–95.7%, 76.5–93.8%), the *S_ens_* values for the endoplasmic reticulum proteins (Endo), nuclear proteins (Nucl), and secreted proteins (Secr) were the highest among all the methods analyzed in this study (Table 3). 

Similarly, based on dataset ZW225, the iAPSL-IF was also compared with seven known predictors available in the literature, and the resulting data were presented in Table 4. The *OA* (97.3%) generated by the iAPSL-IF was higher than most of the methods tested in this study, but was lower than the PSSM-trigram by 0.5%. The *S_ens_* values for the Cyto and Memb proteins identified by the iAPSL-IF were the highest (100% and 98.9%, respectively) among all these methods, but the *S_ens_* for the Mito and Nucl proteins were slightly lower than the PSSM-trigram. Given that the sequence features were also extracted from the PSSM by the PSSM-trigram [7], we concluded that the PSSMs contained important evolutionary information about protein sequences, and were very useful for identifying the subcellular location of APs. 

Taken together, the overall performance of the iAPSL-IF developed in this study was better than the previous methods reported in the literature. It is known that increased or decreased apoptosis is associated with human diseases, and the function of APs is strongly related to their subcellular location. Therefore, the high-throughput and sequence-based iAPSL-IF will benefit researchers by allowing fast and efficient identification of the subcellular location of APs, which could be candidate targets for the development of novel diagnostics, vaccines, and therapeutics for human diseases.

## 3. Materials and Methods

### 3.1. Datasets

In this study, three widely used datasets ZD98, CL317, and ZW225 were used to test the performance of proposed methods for identifying the subcellular location of APs. The protein sequences were retrieved from the SWISS-PROT database (available online: https://www.uniprot.org/uniprot/), a source which includes protein sequences for human and the other organisms (e.g., pig, bovine, rat, chicken, African clawed frog, and fruit fly). The dataset ZD98 contained 98 proteins: 43 Cyto, 13 Mito, 30 Memb, and 12 other proteins [1]. The dataset CL317 consisted of 317 proteins classified into 6 classes: 112 Cyto, 55 Memb, 52 Nucl, 47 Endo, 34 Mito, and 17 Secr [10,11]. The dataset ZW225 contained 225 proteins classified into 4 classes: 89 Memb, 70 Cyto, 41 Nucl, and 25 Mito [2].

### 3.2. Markov Chains 

The Markov chains have a substantial mathematical foundation [21]. Suppose *S* is a set of finite state, *S* = {*S*_1_, *S*_2_, …, *S*_N_}, where *S* is called state set and the symbol *S*_N_ (N is positive integer) is called state. For a random sequence {*X_t_*}*_t_*_=0_, *X_t_* refers to a state in *S* at time *t.* The state of Markov chains is *q_t_* at *t* time, if the state *q_t_*_+1_ at *t* + 1 only related to *q_t_*, i.e.,

$$P\left(X_{t+1}=q_{t+1}|X_{t}=q_{t},X_{t-1}=q_{t-1},...,X_{0}=q_{0}\right)=P\left(X_{t+1}=q_{t+1}|X_{t}=q_{t}\right),$$

In the formula, *q*_0_, *q*_1_, …, *q_n_*∈*S*. Thus, the {*X_t_*}*_t_*_=0_ is called Markov chains [22].

The matrix *M* = {*P_i,j_*} (*i*, *j*∈*S*) is the transition matrix of Markov chains, and $P_{i,j}=P\left(X_{t+1}=j|X_{t}=i\right)$ is the transition probability. *M* can be expressed as:

$$M=\left(\begin{array}{cccc} P_{11} & P_{12} & \cdots & P_{1n}\\ P_{21} & P_{22} & \cdots & P_{2n}\\ \vdots & \vdots & \ddots & \vdots \\ P_{n1} & P_{n2} & \cdots & P_{\mathrm{nn}} \end{array}\right),$$

In this matrix, 0 ≤ *P_i,j_* ≤ 1, $\sum_{j=1}^{N}P_{i,j}=1$ for all state *i*, *j*.

Suppose the length of a protein sequence S is *L*, *A_i_* is the *i*th amino acid of S. Thus, this protein sequence can be represented as *Pro* = *A*_1_ *A*_2_ *A*_3_ *… A_i_ A_i_*_+1_ *… A_L_*. In this study, we analyzed the probability of each amino acid residue affected by the previous amino acid residue, which is expressed as:

$$P\left(A_{i}|A_{i-1}\right)=\frac{F(A_{i-1}A_{i})}{F(A_{i-1})},$$

where *F*(*A_i_*_−1_*A_i_*) and *F*(*A_i_*_−1_) is the frequency of amino acid pairs *A_i_*_−1_*A_i_* and *A_i_*_−1_, respectively. 

Every protein sequence consists of 20 native amino acids, thus, the combinations of amino acid pairs generate a 20 × 20 matrix. In addition, amino acid composition is a basic feature of every protein sequence, which consists of 20 discrete numbers. Each of the numbers represent the frequency of the native amino acid residues in a protein sequence [39]. In this study, every protein sequence was represented by a 420 (20 × 20 + 20) dimensional vector by combining amino acid composition and Markov chains.

### 3.3. Physiochemical Properties of Amino Acids

In this study, 10 physicochemical properties were adopted: polarity, secondary structure, molecular volume, codon diversity, electrostatic charge, hydrophobicity, hydrophilicity, side-chain volume, polarizability, and solvent-accessible surface area, which were represented as P^(1)^, P^(2)^, P^(3)^, P^(4)^, P^(5)^, P^(6)^, P^(7)^, P^(8)^, P^(9)^, P^(10)^, respectively [35]. The original values of these physicochemical properties (Table 5) were normalized by the following formula before use, as described previously [40]:

$$P_{n}^{m}⇐\frac{P_{n}^{m}-P_{n}}{SD(P_{n}^{})}(m=1,2,\ldots ,20;\;n=1,2,\ldots ,10)$$

where $P_{n}^{m}$ is the value of the *n* type physicochemical property of the *m* type amino acid, $P_{n}$ and $SD(P_{n}^{})$ are the mean and standard deviation of the *n* type physicochemical property of the 20 native amino acids. Therefore, a protein sequence with a length of *L* can be encoded into ten different numerical series as follows:

$$\mathrm{Pr}o=\{\begin{array}{cccccccccccc} P_{1}^{\left(1\right)} & P_{2}^{\left(1\right)} & P_{3}^{\left(1\right)} & P_{4}^{\left(1\right)} & P_{5}^{\left(1\right)} & P_{6}^{\left(1\right)} & P_{7}^{\left(1\right)} & P_{8}^{\left(1\right)} & P_{9}^{\left(1\right)} & P_{10}^{\left(1\right)} & \cdots & P_{L}^{\left(1\right)}\\ P_{1}^{\left(2\right)} & P_{2}^{\left(2\right)} & P_{3}^{\left(2\right)} & P_{4}^{\left(2\right)} & P_{5}^{\left(2\right)} & P_{6}^{\left(2\right)} & P_{7}^{\left(2\right)} & P_{8}^{\left(2\right)} & P_{9}^{\left(2\right)} & P_{10}^{\left(2\right)} & \cdots & P_{L}^{\left(2\right)}\\ P_{1}^{\left(3\right)} & P_{2}^{\left(3\right)} & P_{3}^{\left(3\right)} & P_{4}^{\left(3\right)} & P_{5}^{\left(3\right)} & P_{6}^{\left(3\right)} & P_{7}^{\left(3\right)} & P_{8}^{\left(3\right)} & P_{9}^{\left(3\right)} & P_{10}^{\left(3\right)} & \cdots & P_{L}^{\left(3\right)}\\ P_{1}^{\left(4\right)} & P_{2}^{\left(4\right)} & P_{3}^{\left(4\right)} & P_{4}^{\left(4\right)} & P_{5}^{\left(4\right)} & P_{6}^{\left(4\right)} & P_{7}^{\left(4\right)} & P_{8}^{\left(4\right)} & P_{9}^{\left(4\right)} & P_{10}^{\left(4\right)} & \cdots & P_{L}^{\left(4\right)}\\ P_{1}^{\left(5\right)} & P_{2}^{\left(5\right)} & P_{3}^{\left(5\right)} & P_{4}^{\left(5\right)} & P_{5}^{\left(5\right)} & P_{6}^{\left(5\right)} & P_{7}^{\left(5\right)} & P_{8}^{\left(5\right)} & P_{9}^{\left(5\right)} & P_{10}^{\left(5\right)} & \cdots & P_{L}^{\left(5\right)}\\ P_{1}^{\left(6\right)} & P_{2}^{\left(6\right)} & P_{3}^{\left(6\right)} & P_{4}^{\left(6\right)} & P_{5}^{\left(6\right)} & P_{6}^{\left(6\right)} & P_{7}^{\left(6\right)} & P_{8}^{\left(6\right)} & P_{9}^{\left(6\right)} & P_{10}^{\left(6\right)} & \cdots & P_{L}^{\left(6\right)}\\ P_{1}^{\left(7\right)} & P_{2}^{\left(7\right)} & P_{3}^{\left(7\right)} & P_{4}^{\left(7\right)} & P_{5}^{\left(7\right)} & P_{6}^{\left(7\right)} & P_{7}^{\left(7\right)} & P_{8}^{\left(7\right)} & P_{9}^{\left(7\right)} & P_{10}^{\left(7\right)} & \cdots & P_{L}^{\left(7\right)}\\ P_{1}^{\left(8\right)} & P_{2}^{\left(8\right)} & P_{3}^{\left(8\right)} & P_{4}^{\left(8\right)} & P_{5}^{\left(8\right)} & P_{6}^{\left(8\right)} & P_{7}^{\left(8\right)} & P_{8}^{\left(8\right)} & P_{9}^{\left(8\right)} & P_{10}^{\left(8\right)} & \cdots & P_{L}^{\left(8\right)}\\ P_{1}^{\left(9\right)} & P_{2}^{\left(9\right)} & P_{3}^{\left(9\right)} & P_{4}^{\left(9\right)} & P_{5}^{\left(9\right)} & P_{6}^{\left(9\right)} & P_{7}^{\left(9\right)} & P_{8}^{\left(9\right)} & P_{9}^{\left(9\right)} & P_{10}^{\left(9\right)} & \cdots & P_{L}^{\left(9\right)}\\ P_{1}^{\left(10\right)} & P_{2}^{\left(10\right)} & P_{3}^{\left(10\right)} & P_{4}^{\left(10\right)} & P_{5}^{\left(10\right)} & P_{6}^{\left(10\right)} & P_{7}^{\left(10\right)} & P_{8}^{\left(10\right)} & P_{9}^{\left(10\right)} & P_{10}^{\left(10\right)} & \cdots & P_{L}^{\left(10\right)} \end{array}$$

where $P_{1}^{\left(1\right)}$ is the polarity value of the first amino acid in the protein sequence, $P_{2}^{\left(2\right)}$ is the secondary structure value of the second amino acid in the sequence, and so forth.

### 3.4. PSSM

In this study, we analyzed all protein sequences using the PSI-BLAST program [26] against a NR database of the NCBI (available online: https://www.ncbi.nlm.nih.gov/) with default parameters, except the e-value threshold and the maximum number of iterations were set to 0.001 and 3, respectively, as described previously [35]. Each protein sequence generated a corresponding *L* × 20 PSSM as follows:

$$PSSM=\left(\begin{array}{cccc} p_{1,1} & p_{1,2} & \cdots & p_{1,20}\\ p_{2,1} & p_{2,2} & \cdots & p_{2,20}\\ \vdots & \vdots & \ddots & \vdots \\ p_{L,1} & p_{L,2} & \cdots & p_{L,20} \end{array}\right)$$

where *L* is the length of a protein sequence and 20 is the number of native amino acids. The element *p_ij_* represents the occurrence probability of amino acid *j* at position *i* of the protein sequence. The rows of the matrix represent the positions of the sequence, while the columns represent the 20 amino acids [42].

The original values of the PSSM were normalized to reduce the noise and bias using the sigmoid function: f(*x*) = 1/(1 + e^−*x*^) [16], where *x* is the original value of the PSSM. 

### 3.5. ACC Transformation

In this study, the matrices of protein sequences with different lengths were transformed into fixed-length vectors using the ACC method, as described previously [35]. The method has two variables: auto covariance *A*(*j*, *g*) measures the correlation of the same property between amino acids by a distance of *g* along the sequence; cross-covariance *C*(*j*, *k*, *g*) measures different properties [43]. Both variables can be computed using the following formulae:

$$A(j,g)=\sum_{i=1}^{L-g}\left(p_{i,j}-\bar{P_{j}})(p_{i+g,j}-\bar{P_{j}})/(L-g\right)$$

$$C(j,k,g)=\sum_{i=1}^{L-g}\left(p_{i,j}-\bar{P_{j}})(p_{i+g,k}-\bar{P_{k}})/(L-g\right)$$

where $\bar{P_{j}}=\frac{1}{L}\sum_{i=1}^{L}p_{i,j}$ and $\bar{P_{k}}=\frac{1}{L}\sum_{i=1}^{L}p_{i,k}$ represent the average scores of amino acid *j* and *k*, respectively. *L* is the length of the protein sequence, while *j* and *k* represent different amino acids, and *g* is the gap between two amino acids [29].

In this study, for the physicochemical property matrices of protein sequences, the number of auto-covariance variables is 10 × *G*, while the number of cross-covariance variables is 10 × 9 × *G*. Hence, each protein sequence can be encoded by a 100 × *G* dimensional feature vector. Likewise, for the PSSMs of protein sequences, the numbers of auto-covariance variables and cross-covariance variables are 20 × *G*, and 20 × 19 × *G*, respectively. Therefore, every protein sequence can be replaced by a 400 × *G* dimensional feature vector, where *G* is the maximal value for *g*.

### 3.6. SVM and SVM-RFE

In this study, SVM was adopted as the classifier using the LIBSVM algorithm package [44], in which four basic kernel functions are provided: linear, polynomial, Gaussian, and radial basis function (RBF). We chose the RBF as the kernel function, because it had a better boundary response and could reflect the distribution of the dataset more accurately [34]. The two parameters *C* and γ were optimized by the jackknife cross-validation test on the datasets.

In order to decrease feature abundance and computation complexity, we reduced the feature dimensions using the SVM-RFE method [35]. Briefly, a matrix was constructed based on all the feature vectors of protein sequences in a dataset, where each row represented a protein sequence and each column a feature [7]. Then, a ranked feature list was obtained by running the SVM-RFE algorithm based on the feature importance. Subsequently, each protein sequence was encoded by an optimal subset of top-*K* ranked features. 

### 3.7. Performance Measurement

In this study, the jackknife cross-validation test was chosen to measure the performance of predictors, because it is recognized as an objective and rigorous statistical test [45]. In a dataset, one protein sequence was selected as the test set each time, and the remaining were used as the training set. All the protein sequences were selected in turn, and finished until all the sequences were tested. 

Four widely-used measurements, including the *OA*, *S_ens_*, *S_pec_*, and *MCC* were used to measure the predictive capability of the classification, as described previously [45]. They were calculated using the following formulae: *OA* = (*TP* + *TN*)/(*TP* + *TN* + *FP* + *FN*); *S_ens_* = *TP*/(*TP* + *FN*); *S_pec_* = *TN*/(*TN* + *TP*); *MCC* = [(*TP* × *TN*) − (*FP* × *FN*)]/√[(*TP* + *FP*)(*TP* + *FN*)(*TN* + *FP*)(*TN* + *FN*)], where *TP*, *TN*, *FP*, and *FN* represent the numbers of true positive, true negative, false positive, and false negative results, respectively [42].

## 4. Conclusions

In this study, we developed a novel sequence-based method, iAPSL-IF, for the identification of the subcellular location of APs using integrative features captured from Markov chains, physicochemical property matrices, and PSSMs of amino acid sequences. Based on the three datasets ZD98, CL317, and ZW225, the iAPSL-IF outperformed the known predictors reported in the literature for several classes of subcellular location of APs, including the membrane proteins, cytoplasmic proteins, endoplasmic reticulum proteins, nuclear proteins, and secreted proteins. The source codes were written in the programming language Python 3, which is available by contacting the authors. In our future research, a web-based platform will be constructed for further application of the iAPSL-IF.

## Figures and Tables

**Table 1:** Performance of the iAPSL-IF on the three datasets.

Dataset	Location	*S_ens_* (%)	*S_pec_* (%)	*MCC*	*OA* (%)
ZD98	Cyto	97.67	100	0.98	98.98
	Memb	100	98.53	0.98	
	Mito	100	100	1.0	
	other	100	100	1.0	
CL317	Cyto	95.54	97.07	0.92	94.95
	Memb	94.55	98.85	0.93	
	Mito	88.24	98.94	0.88	
	Secr	100	99.67	0.97	
	Nucl	94.23	98.87	0.93	
	Endo	97.87	100	0.99	
ZW225	Cyto	100	98.71	0.98	97.33
	Memb	98.88	99.26	0.98	
	Mito	88.00	99.50	0.90	
	Nucl	95.12	98.98	0.94	

**Table 2:** Performance comparison of different methods on the ZD98 dataset.

Method	*S_ens_* for Each Class (%)				*OA* (%)	Reference
	Cyto	Memb	Mito	Other		
Covariant	97.7	73.3	30.8	25.0	72.5	[1]
ID_SVM	95.3	93.3	84.6	58.3	88.8	[10]
DWT_SVM	95.4	93.3	53.9	91.7	88.8	[26]
ID	90.7	90.0	92.3	91.7	90.8	[11]
EBGW_SVM	97.7	90.0	92.3	83.3	92.9	[2]
PseAAC_SVM	95.3	93.3	92.3	83.3	92.9	[36]
DF_SVM	97.7	96.7	92.3	75.0	93.9	[16]
Dual_layer SVM	95.4	96.7	92.3	91.7	94.9	[37]
APSLAP	95.3	90.0	100	91.7	94.9	[27]
FKNN	95.3	96.7	100	91.7	95.9	[12]
PSSM-AC	97.7	96.7	100	83.3	95.9	[38]
PSSM-trigram	95.3	100	100	91.7	96.9	[7]
iAPSL-IF	97.7	100	100	100	99.0	This study

**Table 3:** Performance comparison of different methods on the CL317 dataset.

Method	*S_ens_* for Each Class (%)						*OA* (%)	Reference
	Cyto	Memb	Mito	Secr	Nucl	Endo		
ID	81.3	81.8	85.3	88.2	82.7	83.0	82.7	[10]
ID_SVM	91.1	89.1	79.4	58.8	73.1	87.2	84.2	[11]
DF_SVM	92.9	85.5	76.5	76.5	93.6	86.5	88.0	[16]
Auto_Cova	86.4	90.7	93.8	85.7	92.1	93.8	90.0	[14]
FKNN	93.8	92.7	82.4	76.5	90.4	93.6	90.9	[12]
PseAAC_SVM	93.8	90.9	85.3	76.5	90.4	95.7	91.1	[36]
EN_FKNN	98.2	83.6	79.4	82.4	90.4	97.9	91.5	[26]
PSSM-AC	93.8	90.9	91.2	82.4	86.5	95.7	91.5	[38]
APSLAP	99.1	89.1	85.3	88.2	84.3	95.8	92.4	[27]
EI_SVM	94.6	95.7	92.7	82.4	90.4	70.6	91.1	[18]
iAPSL-IF	95.5	94.5	88.2	100	94.2	97.9	95.0	This study

**Table 4:** Performance comparison of different methods on the ZW225 dataset.

Method	*S_ens_* for Each Class (%)				*OA* (%)	Reference
	Cyto	Memb	Mito	Nucl		
EBGW_SVM	90.0	93.3	60.0	63.4	83.1	[2]
DF_SVM	87.1	92.1	64.0	73.2	84.0	[16]
PSSM-AC	82.9	92.1	68.0	78.0	84.0	[38]
ID_SVM	92.9	91.0	68.0	73.2	85.8	[11]
Auto_Cova	81.3	93.3	85.7	84.6	87.1	[14]
EN_FKNN	94.3	94.4	60.0	80.5	88.0	[26]
PSSM-trigram	97.1	98.9	96.0	97.6	97.8	[7]
iAPSL-IF	100	98.9	88.0	95.1	97.3	This study

**Table 5:** The original values of the ten physiochemical properties for all amino acids [41].

AA	P^(1)^	P^(2)^	P^(3)^	P^(4)^	P^(5)^	P^(6)^	P^(7)^	P^(8)^	P^(9)^	P^(10)^
A	8.100	−1.302	−0.733	1.57	−0.146	0.620	−0.500	27.500	0.046	1.181
C	5.500	0.465	−0.862	−1.02	−0.255	0.290	−1.000	44.600	0.128	1.461
D	13.000	0.302	−3.656	−0.259	−3.242	−0.900	3.000	40.000	0.105	1.587
E	12.300	−1.453	1.477	0.113	−0.837	−0.740	3.000	62.000	0.151	1.862
F	5.200	−0.59	1.891	−0.397	0.412	1.190	−2.500	115.500	0.290	2.228
G	9.000	1.652	1.33	1.045	2.064	0.480	0.000	0.000	0.000	0.881
H	10.400	−0.417	−1.673	−1.474	−0.078	−0.400	−0.500	79.000	0.230	2.025
I	5.200	−0.547	2.131	0.393	0.816	1.380	−1.800	93.500	0.186	1.810
K	11.300	−0.561	0.533	−0.277	1.648	−1.500	3.000	100.000	0.219	2.258
L	4.900	−0.987	−1.505	1.266	−0.912	1.060	−1.800	93.500	0.186	1.931
M	5.700	−1.524	2.219	−1.005	1.212	0.640	−1.300	94.100	0.221	2.034
N	11.600	0.828	1.299	−0.169	0.933	−0.780	2.000	58.700	0.134	1.655
P	8.000	2.081	−1.628	0.421	−1.392	0.120	0.000	41.900	0.131	1.468
Q	10.500	−0.179	−3.005	−0.503	−1.853	−0.850	0.200	80.700	0.180	1.932
R	10.500	−0.055	1.502	0.44	2.897	−2.530	3.000	105.000	0.291	2.560
S	9.200	1.399	−4.76	0.67	−2.647	−0.180	0.300	29.300	0.062	1.298
T	8.000	0.326	2.213	0.908	1.313	−0.050	−0.400	51.300	0.108	1.525
V	5.900	−0.279	−0.544	1.242	−1.262	1.080	−1.500	71.500	0.140	1.645
W	5.400	0.009	0.672	−2.128	−0.184	0.810	−3.400	145.500	0.409	2.663
Y	6.200	0.83	3.097	−0.838	1.512	0.260	−2.300	117.300	0.298	2.368
